# A dynamic examination of the digital circuit implementing the Fitzhugh-Nagumo neuron model with emphasis on low power consumption and high precision

**DOI:** 10.1371/journal.pone.0327595

**Published:** 2025-08-20

**Authors:** Mehdi Nadiri Andabili, Soheila Nazari, Tohid Moosazadeh

**Affiliations:** 1 Department of Electrical Engineering, Central Tehran Branch, Islamic Azad University, Tehran, Iran; 2 Faculty of Electrical Engineering, Shahid Beheshti University, Tehran, Iran; University of Kashmir, INDIA

## Abstract

Neuromorphic computing has got more attention in various tasks during recent years. The main goal of this field is to explore neural functionality in the brain. The studies of spiking neurons and Spiking Neural Networks (SNNs) are vital to understand how brain-inspired neural system work. In this paper, the modified Fitzhugh-Nagumo (FHN) model is proposed based on Coordinate Rotation Digital Computer (CORDIC) algorithm to emulate biological behaviors of the original neuron model. The presented CORDIC method eliminates multipliers by using adder and shifter operations, which provides efficient digital hardware implementation of the FHN model. Error analysis and dynamic assessments confirm that the CORDIC-based FHN model is capable to follow the biological behaviors of the original model with high accuracy. Additionally, to further check the compatibility of the CORDIC-based FHN model with the original model, chaotic behaviors of both models on bifurcation and maximum Lyapunov exponent diagrams are investigated. Considering that the CORDIC-based FHN model has a high compatibility with the original model, its superiority over the original model is the possibility of hardware implementation with low power consumption. To analyze further, two cost functions are defined based on operation frequency, power, and error to confirm the efficiency of the proposed hardware compared to previous studies. As a result of its low power consumption, minimal error rates, and high-frequency capabilities, the proposed hardware demonstrates effectiveness and utility across a range of applications, including the simulation of learning processes in the nervous system that are based on nonlinear and chaotic behaviors.

## Introduction

Neuromorphic computing, which was presented by Carver Mead, is a highly demanding field among researchers during these years [1]. The studies have focused on understanding and implementing the structure and functions of the brain, especially Central Nervous system (CNS) [2]. In fact, the CNS consists of numerous neurons and synapses, and also there are significant connections between them as a complex network. Thus, Spiking Neural Networks (SNNs) have been introduced for detailed understanding of how the CNS performs and transmits information by spikes. Due to the real biological behavior of neurons, which be mimicked by SNNs, these networks have been more popular than Artificial Neural Networks (ANNs), which are bio-inspired systems [3,4]. Additionally, SNNs are employed in a wide range of practical applications like image classification [5], pattern recognition [6], robotic control [7], and medical diagnosis [8]. Since the neuron is the most fundamental component of the CNS, the behavior of the single neuron is essential.

To study the dynamical spiking behaviors of a neuron, different mathematical models have been proposed for SNNs [9–16]. These models are typically represented by ordinary differential equations (ODEs) with diverse levels of factors, including the computational complexity and the biological accuracy. It should be noted that a trade-off exists between both factors, so the models with more accuracy require more computational capacity. In general, there are two categories for all spiking neurons, including spike-based and conductance-based models [17,18]. For producing a response in conductance-based models, the biological neuron requires high accuracy with a high cost. The most famous model in the family is Hodgkin-Huxley (HH) [9], which employs four differential equations to create all behaviors of the spiking neuron. Although the HH model provides complete details of the neural behaviors by using the high physiological characteristics, simulations of the large-scale neuron population of the model are not applicable. The second group of the spiking neuron models is spike-based models, which are described by fewer biological parameters, so the complexity of them is extremely lower. Hence, one of the popular models in this group is FitzHugh-Nagumo (FHN) [13], which is simplified from the HH model and represented by two differential equations. For considering the FHN model, having only one nonlinear term to capture neural spiking behaviors with less computational complexity is a main reason to apply it in the large-scale networks. Additionally, one of the most efficiency-focused dynamic models is the FHN model because it can reproduce all features of the spiking neuron. Moreover, the model is capable of generating chaotic behaviors in special values of the external stimulus [19]. Thus, the FHN model can be a successful candidate for hardware implementation, especially in the large neuron populations [20].

The notable goal of neuromorphic computing is software and hardware implementation of the SNNs with high speed, low power, effective resources, and high accuracy. For this reason, there are two major methods for hardware implementation of the neuron models:

- The first approach is analog circuits, which employ electrical components to resemble the behaviors of the neuron. The crucial benefits of the method are high performance, well development, and acceptable efficiency. Although the method is applicable based on the aforementioned advantages, it suffers from extended development time and its inflexibility [1,21–23].
- The digital method [1,24,25] is the second approach, which improves the drawbacks of the analog circuits, so it is flexible to implement digital hardware of the neuron models with a short device development time. In addition, the impact of noise and error in this approach is less than in analog circuits. On the other hand, the method requires a large area with more power consumption and also slower computations in comparison to another method. During the last few years, using the digital platforms with reconfigurable features has been highly demanding to develop spiking neuron models in the neuromorphic domain. For this reason, Field-Programmable Gate Array (FPGA) [26,27] has been a popular platform to implement digital hardware in this area. In order to parallel imitate neural behaviors, the FPGA-based spiking neural networks make use of digital computing. Thus, the researchers have been motivated to design digital systems by the FPGA platform because of some notable advantages of it, including high speed, high accuracy, flexibility, routing resources, and re-configurability. In contrast, consuming a large area and high power are drawbacks of the approach [28–30].

To accurately evaluate the FHN neuron model, the dynamical behaviors are explored from different mathematical and quantitative perspectives. The analysis confirms the power of the approximated model to reproduce dynamic behaviors as the original model. Researchers from a variety of fields have been interested in chaos since it is a nonlinear phenomenon [31]. Generally, chaotic systems are complex, unpredictable, and sensitive to initial conditions of the system. Examining and analyzing the chaotic behavior in neuron models is important for a number of reasons. The most significant ones of them are neurological diseases, which are related to chaotic behavior and other abnormalities in neural dynamics. By studying chaos, researchers can also better understand how neurons receive information, adapt to changes, and generate complicated behaviors like learning [32]. Chaos, which emerges when the model is stimulated by an externally periodic current, is one of the main reasons why researchers employ the FHN model. A number of the publications concentrate on using various types of externally applied stimuli, including sinusoidal current [21] and hyperbolic tangent stimulus [33]. In general, when the input parameters are modified, the bifurcation diagram, maximum Lyapunov exponent, phase plane, and time-domain waveforms are utilized to visually define chaotic behaviors in the original and CORDIC FHN models. In this paper, we focus more on the dynamical behavior of the proposed FHN model, especially the chaotic behavior. First, we numerically investigate the dynamical behaviors of both models based on the fixed input current. Next, both models are constructed using the sinusoidal stimulus when the values of the frequency and amplitude are altered. Finally, the proposed and original models are compared based on the simulation [19,34–37].

Several studies have addressed the FPGA-based implementation of the FHN neuron model during these years [20,38–45]. The majority of them proposed multiplierless methods to approximate the polynomial nonlinear term by low-cost and simple operations in the original FHN model. The main goals of the presented papers were improving some limitations of using the FPGA platform to realize the spiking neural models. Thus, increasing speed and accuracy and also reducing hardware resources and power consumption, especially in large-scale digital systems, are significant challenges for FPGA-based digital design. It is considered that each modified model was suggested for addressing some of the challenges. For this goal, the presented model in [40] proposed a FHN model that uses hyperbolic functions to implement the dynamic behaviors of the neuron on the FPGA platform. Although realizing the multiplierless FHN model on FPGA for the first time was the novelty of this work, it was only explored in terms of the accuracy of the neural behaviors and hardware resource costs in comparison with the original model. The modified FHN model in [41] used a Piece-Wise Linear (PWL) function to approximate the polynomial nonlinear term of the original model. In addition, the model employed a single astrocyte [46] with the FHN model to achieve an efficient hardware implementation. In [42], the FHN model was approximated by a special type of hyperbolic function to produce the bursting spiking pattern with acceptable results. However, the speed of this work was compared with the original FHN model. The suggested method in [20] used the sine function to remove the nonlinear term of the FHN neuron model. In order to optimize the hardware implementation cost, it was realized by employing the CORDIC IP-Core generator of the FPGA. It is worth noting that employing IP cores in the FPGA family has restrictions, so the realization of the large-scale digital system may be an overhead cost. Moreover, this work was investigated in three aspects of hardware design, including accuracy, maximum frequency, and power consumption. In [45], the modified FHN model, which used a combination of the Piece-Wise Linear (PWL) technique and a base-2 function, was used to implement efficient digital hardware on the FPGA platform.

This paper presents an FPGA-based hardware implementation of the modified FHN neuron model. For this reason, the CORDIC algorithm [47–49] is applied to replace the cubic-polynomial nonlinear term of the original FHN model with simple operations like Shifts and ADDs. Due to the CORDIC algorithm, the computational hardware cost and power consumption are reduced with increasing the maximum frequency. The notable contribution of this work is employing the CORDIC function to eliminate multipliers in the original FHN model for the first time. Although the paper in [20] presented a simple FHN model based on the CORDIC approach, the hardware implementation of the model was realized by utilizing the CORDIC IP-Core on FPGA. Based on the results, the suggested model reproduces the behavioral dynamics of the neuron, such as chaos, and also it can imitate the original FHN model in various states. Moreover, for further assessments, we introduce two cost functions based on three factors to compare the proposed model with other similar works and the original model. As a result, the high-accuracy and low-power CORDIC-based FHN model will be implemented on the FPGA platform. Moreover, because of using simple operations instead of the multipliers, the speed of the proposed system will be higher than the original FHN model. According to the aforementioned benefits of the proposed model, which is applied in the proposed network, it is capable of realizing the large-scale digital implementation.

The rest of this paper is divided into the following sections. In second section, the original FHN model is explained. The proposed model is introduced based on the CORDIC algorithm in third section. The details of time domain, dynamic analysis, chaotic behaviors, and the proposed network are evaluated in fourth section. The hardware implementation of the modified FHN model with the proposed architecture is presented in fifth section. The result of digital implementation is discussed and compared in sixth section. Finally, the conclusion of the work is covered in seventh section.

## Original FHN model

Differential equations can be used to model and define the activity of neurons by collecting numerous aspects of the neuron’s response to stimuli. One example of such a model is the Fitzhugh-Nagumo (FHN) model [13], which is a prototype of an excitable system. The model is a known spiking neuron that was initially derived from the Hodgkin-Huxley model. The original FHN model is introduced by some parameters, and the dynamical system is represented by the following equations.


$$\left\{ \begin{array}{c} \frac{dV}{dt}=V-\frac{V^{3}}{3}-W+I_{in}\\ \frac{dW}{dt}=\frac{1}{T}(a-bW+V) \end{array}\right.\;$$ (1)

where *V* and *W* represent the membrane potential of the neuron and the membrane recovery variable, respectively. Furthermore, three parameters, *a*, *b*, and *T*, are the control parameters, which are fixed dimensionless parameters of the neuron. Finally, the parameter *I*_*in*_ is an input applied current for the voltage equation. Based on the FHN neuron equations, a nonlinear term can cause some problems for hardware implementation, including diminishing performance and implementation efficiency, reducing frequency or speed of hardware, and rising the number of resources. Consequently, the modified FHN model, which is introduced in the next section, is a way to solve the problems and achieve a system with low cost, high speed, and acceptable performance.

## Proposed model

### CORDIC algorithm

A type of the hardware-efficient algorithms for trigonometric, hyperbolic, and logarithmic functions is the coordinate rotation digital computer (CORDIC) [48]. When implementing the complicated functions, including exponential functions, hyperbolic functions, multiplications, divisions, and square roots, the iterative and rotation-based CORDIC algorithm is a useful option to reduce the complexity of digital hardware requirements. The simplicity of the CORDIC algorithm’s structure, which just needs add and shift operations, is what inspired us to utilize it. The CORDIC is a popular method when there are restrictions on using hardware multipliers to implement neuron models. The CORDIC algorithm is used in two modes, which are rotation and vectoring, and also each of them is calculated by three methods, including circular, linear, and hyperbolic coordinate systems. A general form for the algorithm is as follows:


$$\left\{ \begin{array}{c} x_{i+1}=x_{i}-m\cdot \mu_{i}\cdot y_{i}\cdot \delta_{m.i}\\ y_{i+1}=y_{i}-\mu_{i}\cdot x_{i}\cdot \delta_{m.i}\\ z_{i+1}=z_{i}-\mu_{i}\cdot {\sigma \alpha }_{m.i} \end{array}\right.\;$$ (2)

Where *m* can be chosen as −1, 0, or 1 defines a hyperbolic, linear, and circular coordinate system, respectively. The direction of the iteration is shown by $\mu_{i}$.

### Modified model

To approximate the nonlinear term in the FHN model, we use the CORDIC algorithm to increase accuracy and diminish power consumption in the digital implementation of the model. The nonlinear term can be written in the form


$$\text{CORDIC }(\text{V})=\;V^{3}=\;(V\times V)\times V$$ (3)

**Fig 1:**
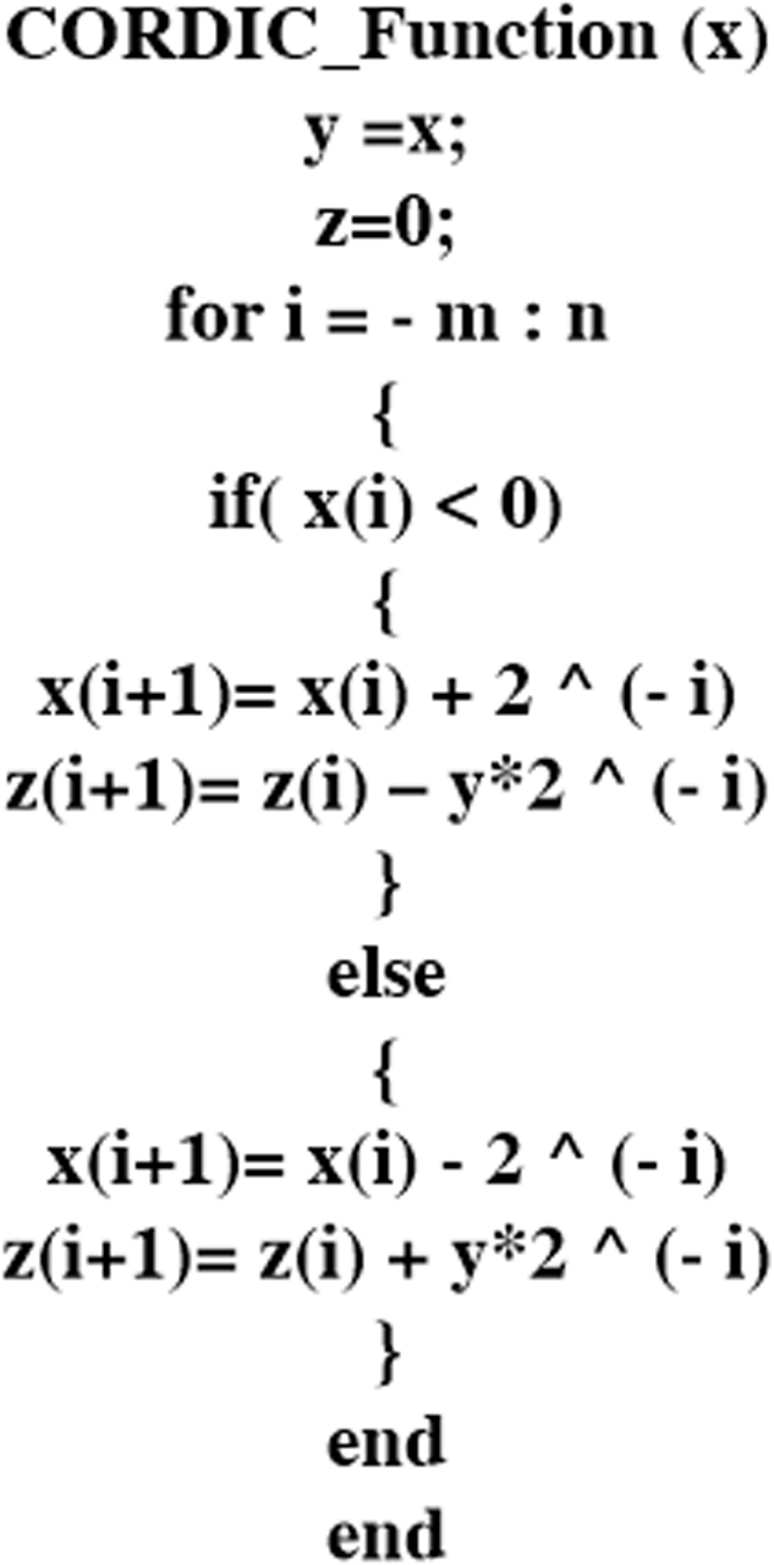
Pseudocode for the CORDIC algorithm to compute multiplication.

**Fig 2:**
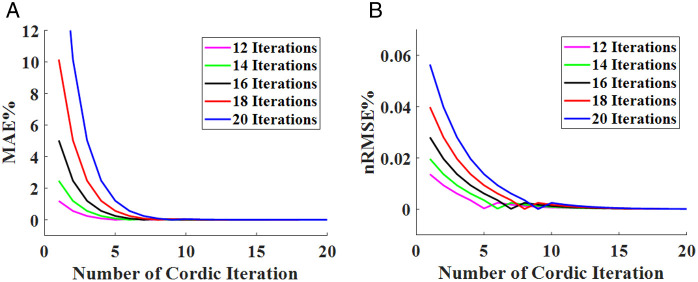
The error plot of the nonlinear term of the FHN model is based on different iteration numbers. (a) MAE. (b) nRMSE.


Which can be simplified by the CORDIC algorithm if using multiplication in two stages. For this reason, we use the multiplication in linear vectoring mode. At the first stage, the multiplication in parentheses is approximated, and then the result of the first stage is multiplied by the CORDIC algorithm. A CORDIC implementation of the multiplication is given by pseudocode, which is shown in Fig 1, where *x*, *y*, *z*, *i*, and *n* represent the multiplicand, the multiplier, the product, the iteration number, and the CORDIC iteration number, respectively. Choosing the number of iterations is the significant part of the CORDIC algorithm because accuracy and resource consumption in digital hardware implementation depend on them. Hence, we calculated two errors, including *MAE* and *nRMSE*, which are introduced in following section, based on different iteration numbers for the nonlinear term of the FHN model in Table 1. This table clearly indicates that reducing the iteration number increases both errors. On the other hand, the accuracy of the algorithm will be high if the number of iterations is chosen to be a big value, but it causes an increase in the number of resources consumed. In addition, we plot both errors, which are illustrated in Fig 2, based on various iteration numbers. Fig 2 demonstrates that a notable drop in *nRMSE* happens when the number of iterations is around 16. Furthermore, we achieved the best number of iterations, which is 16, in each stage. The iteration number in the algorithm starts at −7 and finishes at +8. We reformulate the original FHN model as follows to present the proposed approach.

**Table 1:** Error analysis versus iteration number for the nonlinear term of the FHN model.

Iteration number (each stage)	MAE	nRMSE
50	$2.38\times 10^{-7}$	$2.98\times 10^{-8}$
40	$7.63\times 10^{-6}$	$9.54\times 10^{-7}$
30	$2.44\times 10^{-4}$	$3.052\times 10^{-5}$
20	0.0078	$9.74\times 10^{-4}$
18	0.0156	0.002
16	0.0312	0.0039
14	0.0624	0.0078
12	0.1245	0.0156
10	0.2480	0.031

$$\left\{ \begin{array}{c} \frac{dV}{dt}=V-\frac{CORDIC(V)}{3}-W+I_{in}\\ \frac{dW}{dt}=\frac{1}{T}(a-bW+V) \end{array}\right.\;$$ (4)

**Fig 3:**
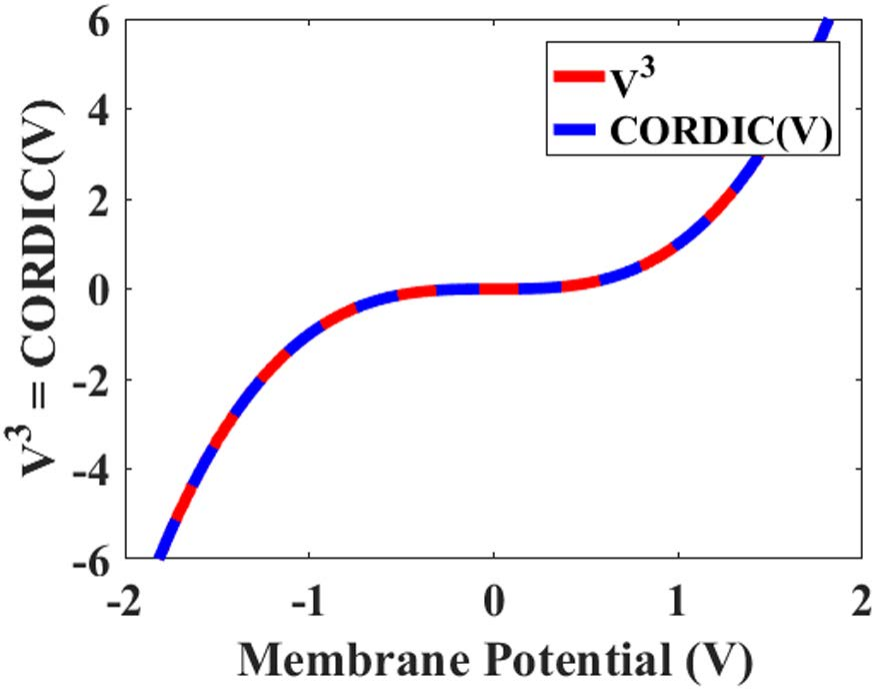
Matching diagram between original nonlinear function ($V^{3}$) and CORDIC function (CORDIC(V)).

Replacing the nonlinear term in the original model, $V^{3}$, with the CORDIC function is the first step to developing the new method. According to Fig 3, polynomial and CORDIC functions display similar characteristics with small error. In fact, creating a new FHN model by applying the method is possible to approximate the original FHN model with the smallest errors. As a result, utilizing the approximation helps us to simplify multiplication in the main model by add and shift operations.

## FHN model validation

To evaluate two FHN models, including the original and proposed models, they must be tested in the time domain by performing dynamical analysis. Additionally, different types of errors must be measured to validate the proposed model.

### Time domain waveforms

Simulation membrane potential results for the original FHN model and the proposed model based on different input currents (*I*_*in*_) and time constants (*T*) are illustrated in Fig 3. They show that the modified model can follow the behaviors of the original model successfully, and also it produces spiking patterns with a high degree of similarity. It is essential to calculate the difference between the proposed and original model when the new model is introduced. Thus, we must measure errors from a spiking timing perspective to compare it with other models. In this section, we explain four equations, which were used a lot in the previous papers, to calculate errors between original and CORDIC-based FHN models.

#### Mean absolute error (MAE).

In this equation, calculating the comparison between the original and CORDIC modes that *MAE* is formulated as follows:


$$MAE=\frac{1}{n}\sum_{i=1}^{n}\left|V_{CORDIC}-V_{FHN}\right|$$ (5)

**Fig 4:**
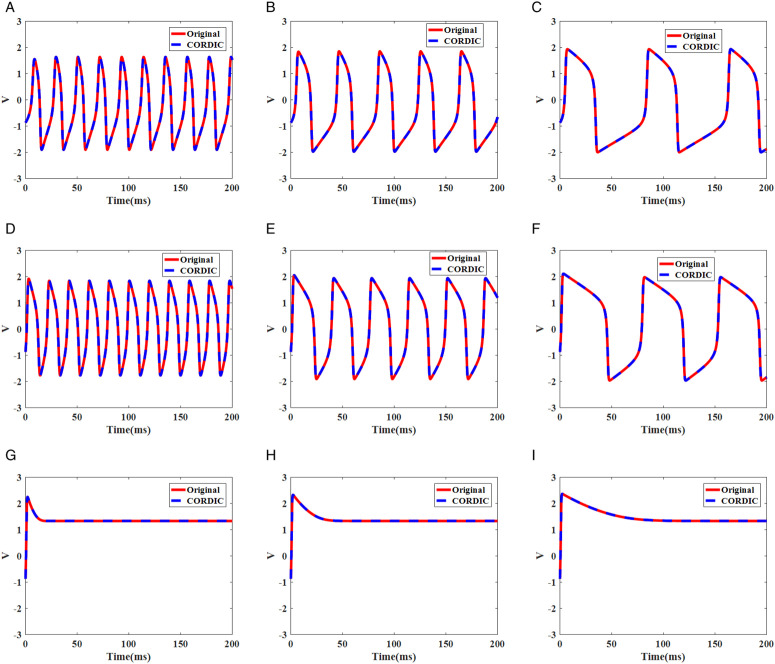
Membrane potential waves in the original and CORDIC-based FHN models according to input current (I_in_) and time constants (T) for (a) I_in_ =0.5 mA and T = 5 ms. (b) I_in_ =0.5 mA and T = 12.5 ms. (c) I_in_ =0.5 mA and T = 30 ms. (d) I_in_ =1 mA and T = 5 ms. (e) I_in_ =1 mA and T = 12.5 ms. (f) I_in_ =1 mA and T = 30 ms. (g) I_in_ =2 mA and T = 5 ms. (h) I_in_ =2 mA and T = 12.5 ms. (i) I_in_ =2 mA and T = 30 ms.

Where *n*, *V*_*FHN*_,and *V*_*CORDIC*_ are the discrete points and the membrane potential values for the original and CORDIC-based FHN models, respectively (Fig 4).

#### Root mean square error (RMSE).

*RMSE* computes the average of squared discrepancies between original and CORDIC FHN models. In fact, the average error magnitude is determined by *RMSE*, which has the following equation:


$$RMSE=\sqrt{\frac{\sum {\mathrm{\backslash nolimits}}_{i=0}^{n}{\left(V_{CORDIC}-V_{FHN}\right)}^{2}}{n}}$$ (6)

#### Normalized root mean square error (nRMSE).

Another error analyzer is nRMSE, which can calculate the variance response between the original and CORDIC FHN models. It is given as below:


$$nRMSE=\frac{1}{V_{\max}-V_{\min}}\sqrt{\frac{\sum {\mathrm{\backslash nolimits}}_{i=0}^{n}{\left(V_{CORDIC}-V_{FHN}\right)}^{2}}{n}}$$ (7)

Here*, V*_*max*_ and *V*_*min*_ are the maximum and minimum values of membrane potential in the original FHN model.

#### Correlation (Corr).

*Corr* is a criterion that can be used to assess a static relationship based on dependency between two models. This error is defined as


$$Corr(V_{CORDIC},V_{FHN})=\frac{COV(V_{CORDIC},V_{FHN})}{\sigma_{CORDIC}.\sigma_{FHN}}$$ (8)

Where *COV* and σ are covariance and standard deviation, respectively. The Corr value should be close to one because it shows that there is high dependence between both models. It should be pointed out that the value of errors must be small. It means that the spike patterns of V_FHN_ and V_CORDIC_ will become more similar when error values drop. For more verification of our suggested model, we calculate four errors, including *MAE*, *RMSE*, *nRMSE*, and *Corr*, in Table 2. Furthermore, Table 2 demonstrates a comparison between types of presented FHN models based on the error levels. As can be seen in the table, the error values in our model are lower than in other similar works. Thus, the CORDIC-based FHN model is more accurate than other previous models based on error levels in various criteria.

**Table 2:** Error analysis between our work and other similar works.

Reference	RMSE	NRMSE%	MAE	Corr%
[41]	0.32	3.42	0.04	NA
[42]	0.47	1.25	0.045	82.5
[20]	0.057	1.43	0.015	NA
[45]	0.019	0.4	0.0034	99.8
**This work**	0.017	0.043	0.00083	99.99

### Dynamical analysis

In this part, we investigate the dynamical analysis between original and CORDIC models to compare more the similarity between them by applying fixed stimulus. In fact, the interactions between nullclines are extremely important when dynamical behaviors are explored. There are two nullclines, which include *V* and *W*, in the FHN model. Computing the equilibrium points is the first step in the analysis. When two equations of the model are equal to zero, the fixed points are obtained. The nullclines of the FHN model are given as the following equations:


$$\frac{dV}{dt}=G(V,W)\Rightarrow \frac{dV}{dt}=G(V,W)=0$$ (9)

$$\frac{dW}{dt}=H(V,W)\Rightarrow \frac{dW}{dt}=H(V,W)=0$$ (10)

Thus, the equations of the original and CORDIC-based FHN models can be written as


$$\left\{ \begin{array}{c} V=\frac{V^{3}}{3}-W+I \end{array}\right.\;$$ (11)

$$\left\{ \begin{array}{c} V=\frac{CORDIC(V)}{3}-W+I \end{array}\right.\;$$ (12)

For the bifurcation analysis, the Jacobian matrix and eigenvalues must be calculated as


$$J(V,W)=[\begin{array}{cc} A & B\\ C & D \end{array}]$$ (13)

Where the coefficients of the Jacobian matrix are given by the following equations:


$$\left\{ \begin{array}{cc} A=\frac{\partial G}{\partial V}, & B=\frac{\partial G}{\partial W}\\ C=\frac{\partial H}{\partial V}, & D=\frac{\partial H}{\partial W} \end{array}\right.\;$$ (14)

**Fig 5:**
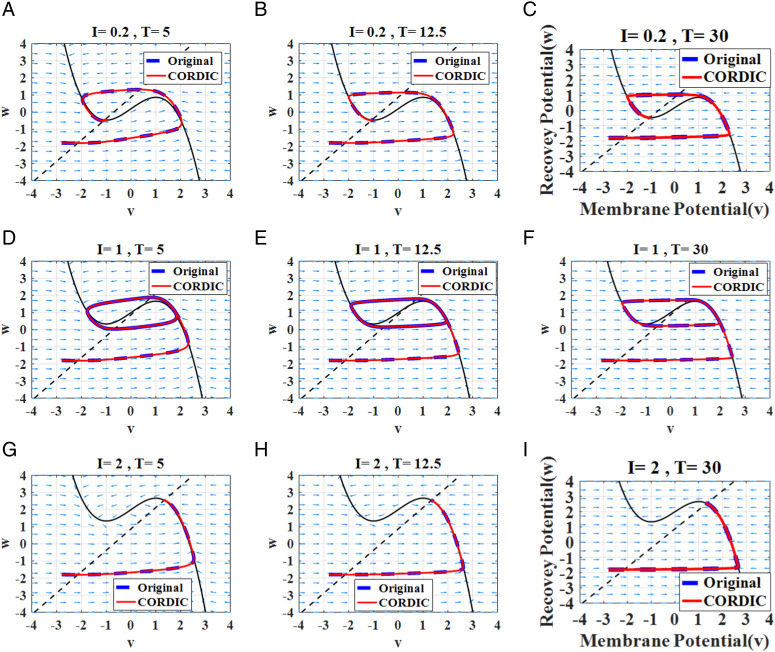
V.*W* phase portraits for the original and proposed FHN model for various input currents (*I*_*in*_) and time constants (*T*).

**Fig 6:**
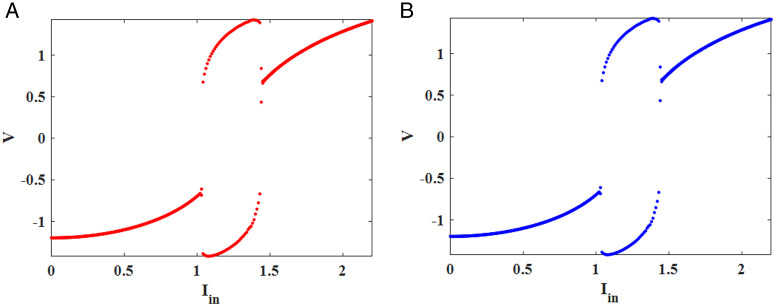
The bifurcation plot of the membrane potential(*V*) based on varying input currents. (a) Original FHN model. (b) Proposed FHN model.

For determining the stability and types of equilibrium points, the determinant (Δ) and time constant (τ) of the Jacobian matrix must be acquired. Furthermore, this τ is the sum of *A* and *D* in the Jacobian matrix. In terms of eigenvalues, there are four states of fixed points, including spiral source, nodal sink, spiral sink, and saddle point. The sign of τ plays a key role in recognizing the stability of the points. The system will be stable if τ is less than zero, and unstable otherwise. Table 3 depicts the numerical results of the dynamical analysis according to the Equations (9)–(14) at varying input currents. From Table 3, it is clear that the proposed method closely follows the original FHN in terms of the equilibrium points, Jacobian matrix values, and the types of their behaviors. The *V-W* phase portrait of both the original and CORDIC-based FHN models is shown in Fig 5. From the trajectory diagrams, it can be seen that the behaviors around the equilibrium points are similar at various input currents. For further considering the dynamic analysis, we plot the Hopf bifurcation, which occurs around the fixed point if a certain parameter changes the stability of the equilibrium point. The Hopf bifurcation plot of the original and proposed FHN model is shown in Fig 6. According to Fig 6, a limit cycle appears around the fixed point when the input current varies from *0.5 mA* to *1.5 mA*. Additionally, both the original and proposed FHN models have the same behavior in the plot. Therefore, the excitability of the neuron is not altered by applying the proposed model to implement the FHN neuron.

**Table 3:** Details of the dynamic analysis in the original and proposed FHN Models based on the different input currents.

Input Current (mA)	FHN Model	Fixed Point	A	B	C	D	Type
**0.5**	Original	(−0.8048, −0.1311)	0.3522	−1	0.08	−0.0640	Stable Spiral
	Proposed	(−0.7344, −0.1035)	1.3919	−1	0.08	−0.0640	Stable Spiral
**1**	Original	(0.4089, 1.3861)	0.8328	−1	0.08	−0.0640	Unstable Node
	Proposed	(0.3906, 1.3711)	0.9416	−1	0.08	−0.0640	Unstable Node
**2**	Original	(1.3341, 2.5426)	−0.7798	−1	0.08	0.064	Stable Node
	Proposed	(1.0937,2.6585)	−0.2991	−1	0.08	0.064	Stable Node

### Dynamical behaviors of the periodic input current

This section examines how the dynamics of the original FHN neuron model and the proposed model are affected by periodic stimulation. There are a number of techniques that can be used to determine whether the FHN model displays chaotic behavior. The time series of the system’s state variable (*V* and *W*) can be seen by performing numerical simulations of the FHN mode equations with a variety of settings. If aperiodic long-term or periodic-doubling behavior is found in the time series, it can be a sign of chaotic behavior. Additionally, the Lyapunov exponent map and bifurcation diagram are the most significant methods to define a chaotic dynamic [31,36,37]. Finally, phase plane analysis is useful to find the chaos in the FHN model. Therefore, we utilize the sinusoidal input current as an external stimulus to analyze the behavior of the FHN model, which can be written as


$$I=I_{in}\cdot Sin(2\pi ft)$$ (15)

where *I*_*in*_ and f refer to the amplitude and frequency of the input stimulus, respectively. During the analysis of the system, varying both *I*_*in*_ and *f* will be used to present the simulation results. It is important to note that the *Ode45* algorithm is applied to solve the equation in Matlab software, and also the initial condition is equal to zero for both variables.

#### 1D-bifurcation diagram.

**Fig 7:**
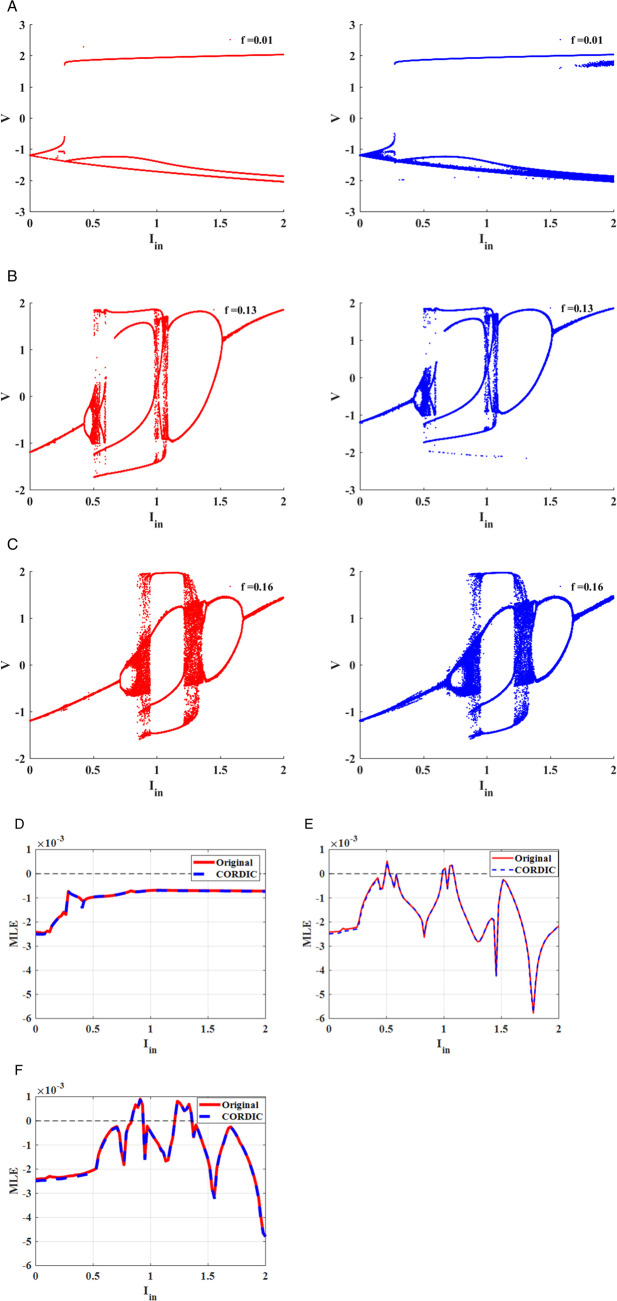
The 1D bifurcation diagrams and *MLE* plots for the original (red) and CORDIC-based FHN (blue) models according to the variations of amplitude *I*_*in*_ in *0* to *2 mA* for sinusoidal input current (*a, d*) *f = 0.01 Hz*. (*b, e*) *f = 0.13 Hz*. (*c, f*) *f = 0.16 Hz.*

**Fig 8:**
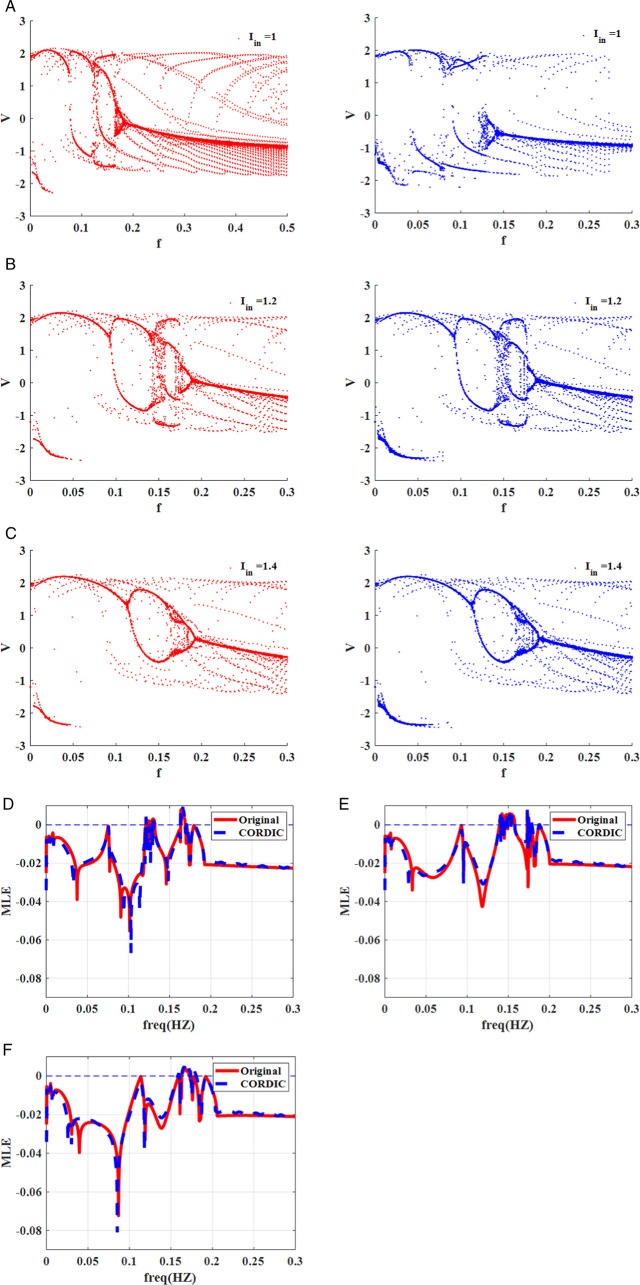
The 1D bifurcation diagrams and *MLE* plots for the original (red) and CORDIC-based FHN (blue) models according to the variations of frequency *f* in *0* to *0.3 Hz* for sinusoidal input current (*a, d*) *I = 1 mA*. (*b, e*) *I = 1.2 mA*. (*c, f*) *I = 1.4 mA.*

A bifurcation diagram is an effective tool for visualizing how a qualitative behavior of the nonlinear system varies when a parameter is changed [21,36,37]. Based on the diagram, some significant data, including stability, period-doubling, periodic window, and chaos, are extracted and visible. To evaluate both the original and CORDIC-based FHN models based on the 1D bifurcation diagram, the frequency and amplitude of the external stimulus are selected as *f* = *0* to *0.3 Hz* and *I*_*in*_ *= 0*–*2 mA*, respectively. Fig 7 exhibits the bifurcation diagram for three different frequency values when the amplitude (*I*_*in*_) of sinusoidal current input is changed from *0* to *2 mA*. The period-doubling behavior is produced for *f* *=* *0.01* because *2*-period and *4*-period cycles are seen in Fig 7a. Furthermore, there are chaotic behaviors for *f* *=* *0.13* and *f* *=* *0.16 Hz* when the parameter *I*_*in*_ is varied (Figs 7b and 7c). In Fig 8, the bifurcation plot for three amplitude values (*I*_*in*_ *= 1, 1.2*, and *1.4 mA*) is displayed when the frequency is varied from *0* to *0.3 Hz*. Chaotic behaviors are limited to a particular frequency range based on the plot. for *I*_*in*_ *= 1* and *1.2 mA*. By comparison, the bifurcation diagrams in both original and proposed FHN models show that the CORDIC-based FHN model accurately produces behaviors that are similar to the original model.

#### Lyapunov exponent map.

The Lyapunov exponent is one of the most helpful metrics for describing periodic and chaotic attractors. The method describes how the system is sensitive to dependence on initial conditions. The Lyapunov exponent for a 1D system is defined by the following equations:


$$F(x+t)-F(x)=\left|\delta_{n}\right|=\left|\delta_{0}\right|.e^{\lambda n}$$ (16)

$$\lambda =\frac{1}{n}\ln\left|\frac{\delta_{n}}{\delta_{0}}\right|$$ (17)

$$\lambda =\lim_{n\to \infty }\left\{\frac{1}{n}\sum {\mathrm{\backslash nolimits}}_{i=0}^{n-1}\ln\left|f\prime (x_{i})\right|\right\}$$ (18)

Where $\delta_{0}$, $\delta_{n}$, and λ are initial separation, separation after *n* iterations,and Lyapunov exponent, respectively. Generally, there are two methods to calculate the Lyapunov exponent, and the Wolf algorithm is a popular technique, especially helpful in chaotic systems. It follows the motion of neighboring trajectories by using the Jacobian matrix of the system to determine the Lyapunov exponent based on how quickly the trajectories diverge or converge. Consequently, we can obtain significant data from the Lyapunov exponent (λ) to accurately analyze the behavior of the dynamical system. If the λ<0, the fixed points are stable, but the period-doubling behavior occurs in the λ=0. Finally, the chaotic behaviors are seen in the dynamical system when the λ>0 [21,31]. In this part, we apply the Wolf algorithm to calculate and plot the maximum Lyapunov exponent (*MLE*) for the original and CORDIC-based FHN models. Fig 7d–7f shows the *MLE* map for three different frequency values when the amplitude (*I*_*in*_) of sinusoidal current input is changed from *0* to *2 mA*. Moreover, in Fig 8d–f, the *MLE* plot for three amplitude values is indicated for changing the frequency from *0* to *0.3 Hz*. Despite having plots that are similar in both Figs 7 and 8, there are small differences between the two models. hence, the errors for some values of *I*_*in*_ and *f* between the two models are computed using the following formula:


$$nRMSE\_MLE=\frac{1}{MLE_{\max}-MLE_{\min}}\sqrt{\frac{\sum_{i=0}^{n}{\left(MLE_{CORDIC}-MLE_{FHN}\right)}^{2}}{n}}$$ (19)

Tables 4 and 5 illustrate the results of calculating the errors for Figs 7 and 8, respectively. By raising the number of iterations, the errors are reduced from the tables, so the *MLE* map of both models is significantly impacted by the number of iterations. In fact, the main reason for this difference between them is the number of iterations in the CORDIC algorithm, which is what distinguishes the original and perturbed initial conditions at the start of the Lyapunov exponent algorithm. Consequently, in disregard of the small variation between models, the modified FHN model has similar behaviors to the original model.

**Table 4:** Error analysis versus iteration number in each stage for the MLE in Fig 6.

Iteration number (each stage)	nRMSE_MLE %		
	f = 0.01 HZ	f = 0.13 HZ	f = 0.16 HZ
14	2.3765	0.7348	0.8333
16	2.3761	0.7345	0.8332
18	2.3751	0.7343	0.8329

**Table 5:** Error analysis versus iteration number in each stage for the MLE in Fig 7.

Iteration number (each stage)	nRMSE_MLE %		
	I_in_ = 1 mA	I_in_ = 1.2 mA	I_in_ =1.4 mA
14	5.2840	5.9590	4.9105
16	5.2821	5.9562	4.9086
18	5.2548	5.9555	4.9064

#### Phase plane and time-domain membrane potential signals.

**Fig 9:**
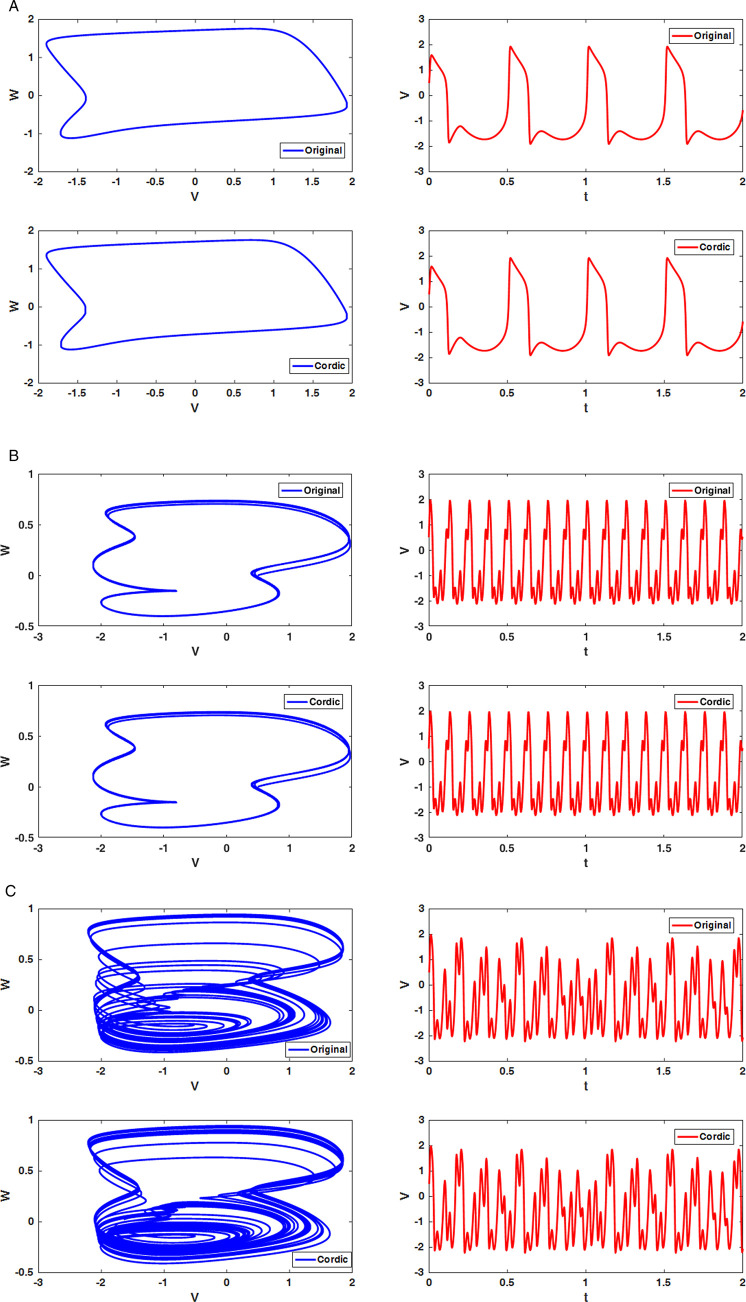
Phase plane (blue) and time-domain spikes of membrane potential (red) for various input currents for both original and CORDIC FHN neuron models. (a) *I*_*in*_ *= 1 mA*, *f = 0.01 Hz*. (b) *I*_*in*_ *= 1 mA*, *f = 0.16 Hz*. (c) *I*_*in*_ *= 1 mA*, *f = 0.13 mA*.

The last method to visually identify chaos in the dynamical system is using the phase plane based on the membrane and recovery voltages. Additionally, applying the time-domain plot for membrane potential is the simplest technique to evaluate the behaviors of the system. Fig 9 illustrates phase plane and time-domain waveforms for the original and modified FHN models for various amplitude and frequency values of external sinusoidal current. When *f* *=* *0.01 Hz* and *I*_*in*_ *=1 mA* are selected, the periodic spiking behaviors are seen for both plots in Fig 9a. Furthermore, chaotic behaviors emerged when *f* *=* *0.13 Hz* and *I*_*in*_ *= 1 mA* were chosen for the external stimulus parameters (Fig 9b). Finally, the period-doubling spikes are generated if the sinusoidal input applies the *f* *=* *0.16 Hz* and *I*_*in*_ *= 1 mA* (Fig 9c). Thus, Fig 9 demonstrates that both original and CORDIC-based FHN models produce similar phase planes and time-domain signals.

According to simulation results of the bifurcation diagrams, maximum Lyapunov exponents, phase planes, and time-domain waveforms for both original and proposed FHN models in the presence of periodic stimulation, the CORDIC-based FHN model is able to completely follow the dynamical behaviors of the original FHN model. Therefore, the suggested model is an appropriate candidate for applying in hardware implementation because all multiplications are eliminated.

### Network activity

**Fig 10:**
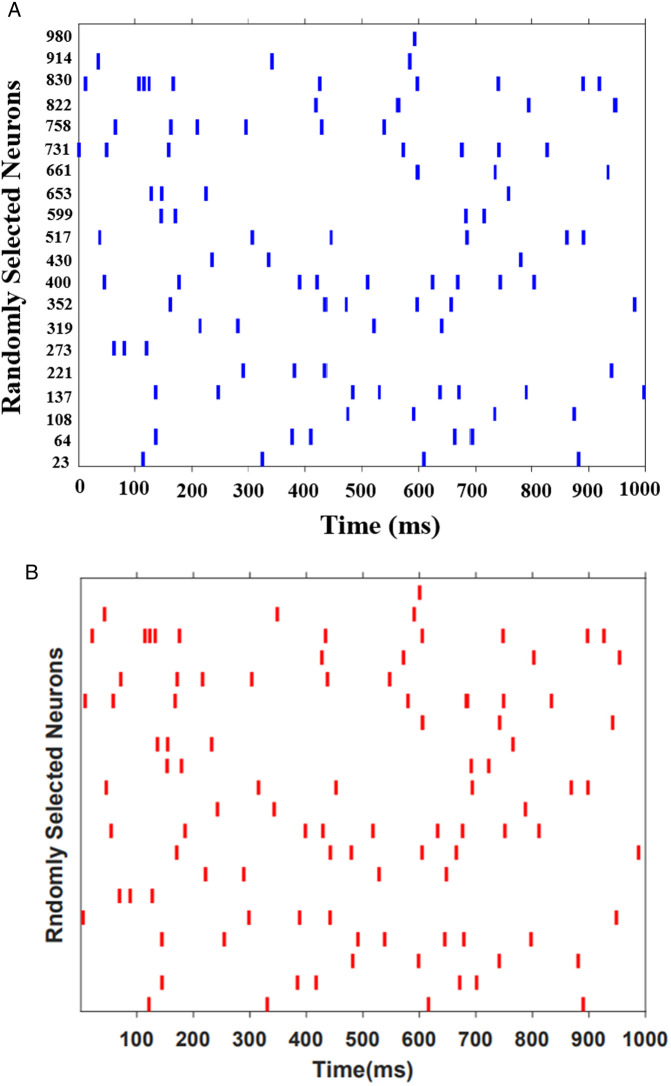
Raster plots illustrate the function of the network with applied input current (*I*_*in*_ *= 0.5 mA*) based on FHN neurons. (a) original. (b) proposed.

In this part, we generate two networks of 1000 randomly connected original and approximated FHN neurons to assess the efficiency of the CORDIC-based FHN neuron in large-scale networks. In both populations, neurons are linked randomly with a probability of *0.1* percent (*0.1%*). It means that each neuron in the networks has a random synaptic connection to *100* other neurons. Furthermore, it is estimated that *80%* of neurons function as excitatory, while the remaining *20%* serve an inhibitory role. In order to evaluate the performance of the proposed networks, an input current of *0.5 mA* is applied to the networks. Furthermore, the duration of the simulation phase was *1200* milliseconds. It is important to mention that the last *1000* milliseconds were cropped for analysis. Fig 10 depicts the raster plot of the simulations of the networks in Matlab. The mean time discrepancy between all spike trains of the original model and the approximate model is *5* milliseconds. It is evident from this figure that the network behaviors of the CORDIC and original FHN models follow each other. In fact, Fig 10 signifies a shared adaptive behavior between the original and approximate models. Therefore, the suggested network can function as a component of spiking neural networks (SNNs), which are utilized in particular applications.

## FHN model implementation

This section describes a digital hardware implementation of the CORDIC-based FHN model by using FPGA. The hardware employs ADD and Shift operations to eliminate multipliers completely.

### Equation discretizing

The first step to implement the neuron model is discretizing the equations, especially variables. For this reason, the Euler method, which is an approach to perform discretization operations, is applied to achieve the low-cost hardware in the FPGA boards. The Equations (1) and (4) are discretized by the Euler technique as below:


$$\frac{du}{dt}=Z(t)$$ (20)

$$\frac{u[n+1]-u[n]}{dt}=Z(t)\to u[n+1]=u[n]+dt.Z(t)$$ (21)

Where both *u* and *Z* are variables and also dt is a time step, which is an essential parameter for the discretization operation. For digital computation, the equations of the original and proposed models must be discretized by Euler’s method as


$$\begin{array}{c} Original\\ FHN \end{array}\left\{ \begin{array}{c} V[n+1]=V[n]+dt.(V[n]-\frac{V^{3}}{3}-W+I_{in})\\ W[n+1]=W[n]+\frac{dt}{T}.(a-b.W[n]+V[n]) \end{array}\right.\;$$ (22)

$$\begin{array}{c} CORDIC\\ FHN \end{array}\left\{ \begin{array}{c} V[n+1]=V[n]+dt.(V[n]-\frac{CORDIC(V[n])}{3}-W+I_{in}) \end{array}\right.\;$$ (23)

**Fig 11:**
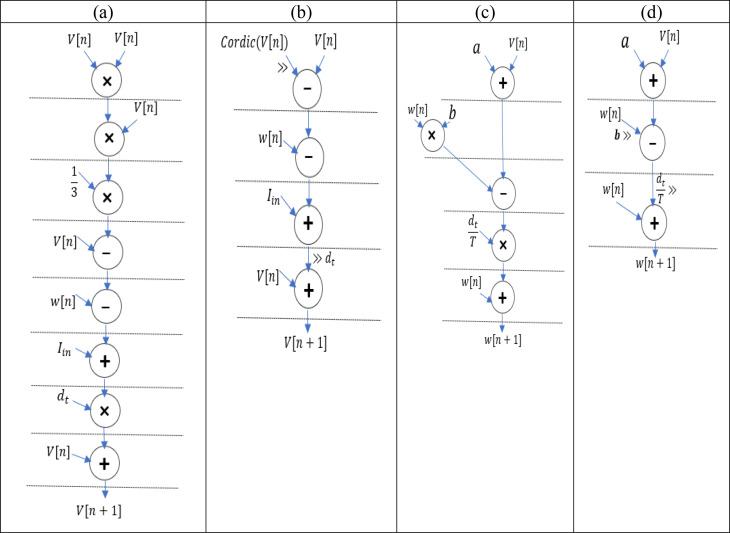
The scheduling diagram of the original and CORDIC FHN models.

In the upper equations, we assess the different values of the step time (*dt*) to realize the best match between both models. Thus, the dt is selected by $\;\frac{1}{32\;}\;$,which is equal to *5* right shifts in the whole equation. The scheduling diagram of the original and modified FHN models is indicated in Fig 11. Based on the figure, the block *a* computes the original membrane potential by using four multipliers. To achieve the multiplierless implementation, the fixed parameters multiplication must be replaced by shift operators. For the goal, the block *b* utilizes the CORDIC function and arithmetic right shift operations to eliminate multipliers in the proposed model equation. The block *c*, which includes two multipliers, calculates the original membrane recovery variable. The last block represents a diagram based on the right shift operations instead of multipliers. It is noteworthy that we just require right shifters for avoiding the multipliers in the proposed model because the fixed parameter multiplications are fractions. As a result, the CORDIC-based FHN model can be implemented multiplierless by using shift operations to reach the low-cost and high-speed hardware.

### Optimum bit-width

Generally, fixed-point form, which is a method of displaying numbers in digital systems, is applied in implementation by FPGA. For some important limitations, such as speed and resources in digital design by FPGA, the fixed-point arithmetic is the best choice to work them out. Therefore, fixed-point form utilizes low digital resources and raises speed in comparison with floating-point arithmetic during the implementation of the digital hardware. Determining the bit width of digital hardware is an essential step to achieve an efficient and low-cost digital system. For this reason, the range of whole variables and parameters should be calculated, and also considering the maximum shift operations is necessary. For the CORDIC-based FHN model, the range of *V*, which is a membrane potential, is *−2* to *+2*. Also, membrane recovery, *W*, is within the range of *−0.5* to *1.5*. The other constant parameters contain *a*, *b*, and *T*, whose values are *0.8*, *0.7*, and *12.5*, respectively. It is noticeable that the parameter *T* is written as $\frac{1}{T}$ in the model, so the value of it is *0.08*. Therefore, they require at least *3* bits by considering a sign bit. Additionally, the CORDIC part needs to take the left shift into account instead of multiplication operations. The maximum left shift is obtained four times. Based on the calculation, the integer part requires *7* bits. On the other hand, the right shift operator is used to compute fraction parts for removing whole multiplications. The maximum right shift is selected by *8* to consider the value of the parameters, variable, and CORDIC part. To prevent any overflow during implementations of the digital hardware, we must regard the extra bit. Finally, the bit width of the digital system is assumed to be *16* bits, which includes *8* bits for the integer part and *8* bits for the fraction part. It should be noted that the digital system is realized based on fixed-point form with *16* bits for all parts of the system.

### Proposed hardware architecture

**Fig 12:**
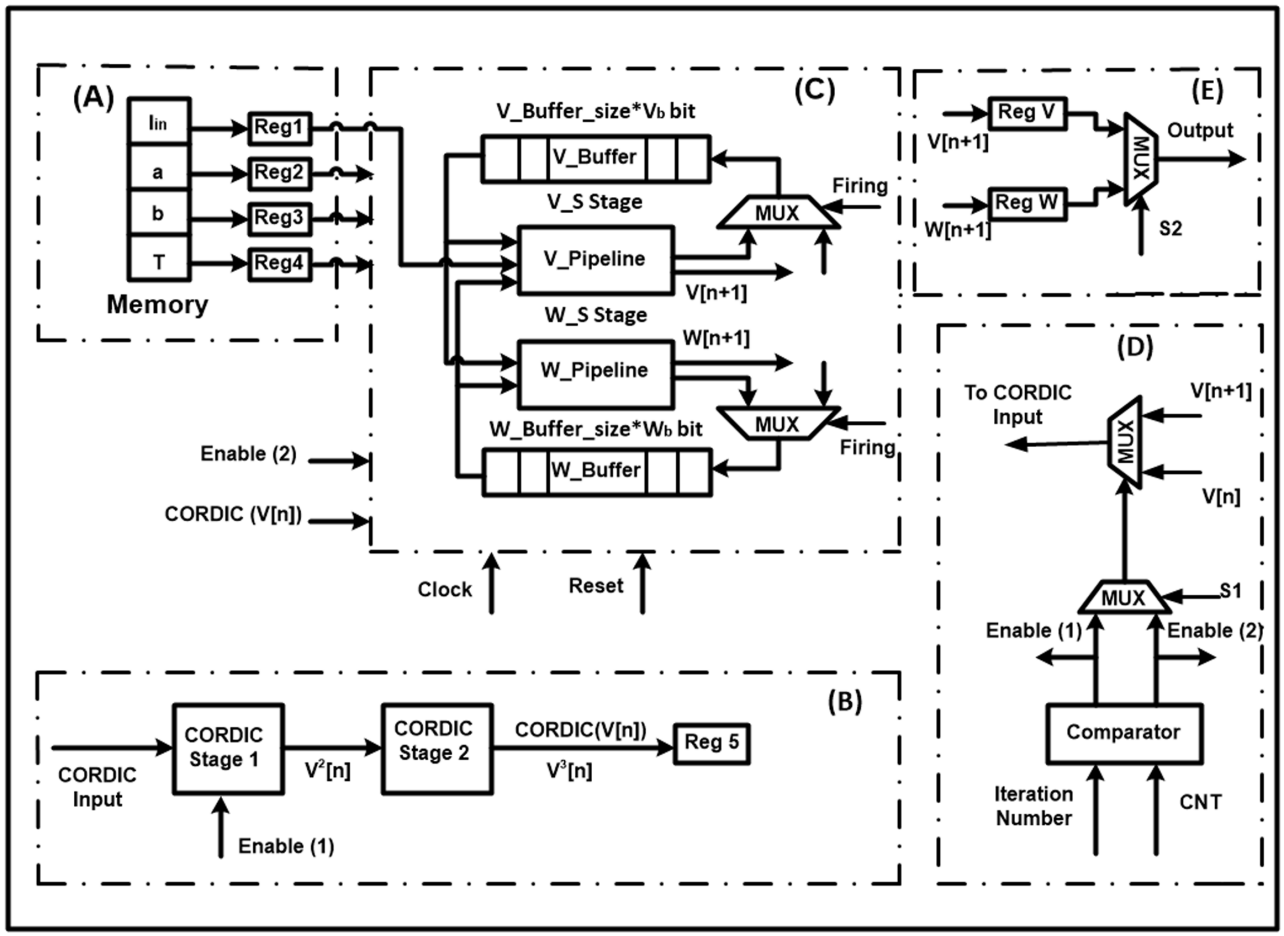
The proposed architecture. (A) Input Unit. (B) CORDIC Unit. (C) Pipeline Unit (D) Output Unit. (E) Control Unit.

The proposed architecture, which is shown in Fig 12 includes five main blocks. It is designed according to the CORDIC-based FHN model formulas presented in Equation (4). The names of the blocks *A*, *B*, *C*, *D*, and *E* are input unit, CORDIC unit, pipeline unit, output unit, and control unit, respectively. The function of each block is explained with details in subsections.

#### Input unit.

The block *A* produces essential data for the next block to activate the FHN neurons. For this reason, we transfer the fixed parameters and input current of the proposed model to registers.

#### CORDIC unit.

**Fig 13:**
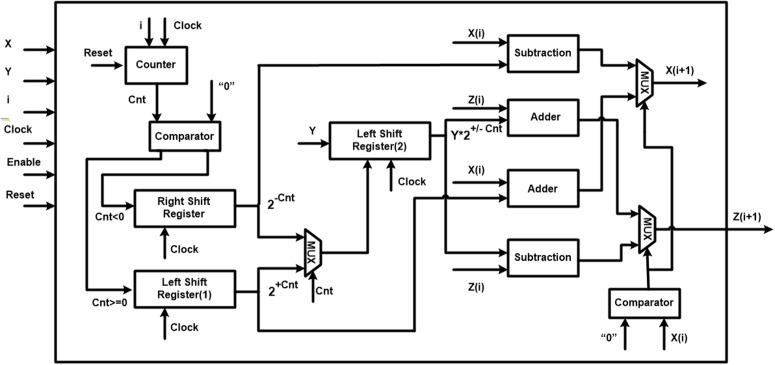
The proposed CORDIC architecture.

The block *B* consists of two CORDIC stage parts, whose details are depicted in Fig 13. The main duty of the unit is calculating the nonlinear term of the original FHN model based on the CORDIC function without utilizing multipliers. The input signal of the unit is *V*, a variable that will be produced by the pipeline unit. After entering the signal to the CORDIC stage *1*, the term $V^{2}$ emerges in the output of the first part. Next, employing the proposed method in the CORDIC Stage 2, the term $V^{3}$ is generated. Finally, the data, which is stored in the register (Reg5), will be transferred to block C.

#### Pipeline unit.

The block *C* generates the final signals of the CORDIC-based FHN model for the output unit based on the two previous blocks. The main reason for using the pipeline technique is increasing the speed for implementing digital hardware of the proposed model. For storing the new data of variables *V* and *W*, two *V_Buffer* and *W_Buffer* buffers are applied. In this unit, the *V_Pipeline* is implemented in the *V_S stage*, and also the *W_Pipeline* is realized in the *W_S stage*. Additionally, the sizes of the *V* and *W* states are represented by *V_Buffer_size* and *W_Buffer_size*, respectively. The values of the buffers are updated by shifting in each clock. Required precision is a significant factor in choosing the *V_Pipeline* and *W_Pipeline*, so the bit-width of the buffers’ unit is equal to the *V_Pipeline* and *W_Pipeline*. Also, for having a high performance of the pipeline structure, the *V* and *W* formations must be synchronized. Thus, the vital conditions and the number of neurons are given as:


$$\left\{ \begin{array}{c} N=V\_Buffer\_Size+V\_S=W\_Buffer\_Size+W\_S\\ V\_Buffer\_Size=W\_Buffer\_Size\\ V\_S=W\_S \end{array}\right.\;$$ (24)

#### Output unit.

This is the block *D* that acquires essential data from the previous block. In fact, the data includes the values of the *V* and W variables, which were generated by the Pipeline unit. The multiplexer (*MUX*) checks the selection gate after storing the data in two registers (Reg *V* and Reg *W*). If the process is completed, the final waves emerge in the output of the block. Otherwise, the *V* and *W* signals are transferred to blocks *E* and *C*, respectively.

#### Control unit.

The major block of the architecture is the block *E*, which controls the other units by generating the required signals. In the unit, two iteration numbers and *CNT*, which are defined in Fig 13, are compared. If the *CNT* is less than the iteration number, the CORDIC unit is active. Otherwise, the pipeline unit is selected. Indeed, the CORDIC input is included V[n+1] when the number of iterations is equal to *CNT*. It should be noted that the signal V[n+1] is provided by the Pipeline unit.

Based on the two CORDIC stages of the proposed architecture in Fig 12, it is essential to observe the completed details of them. Thus, more details about how the CORDIC block, which is applied for both CORDIC stage *1* and CORDIC stage *2*, is implemented can be seen in Fig 13. In the figure, the signals *X, Y, i,* and Z(i+1) denote the multiplication, multiplier, iteration number, and product, respectively. First, the up-counter is responsible for counting the iteration of the CORDIC algorithm and producing the numbers, which are saved in the *CNT* variable. There is a comparison between the *CNT* and the zero number to activate one of the shift registers. The first shift register on the left will be activated if the *CNT* is less than zero, and the right shift register will be chosen otherwise. Following that, a number is generated by the second left shift register using the output of the previous shift register and the *Y* variable. After that, they are transferred to adder and subtractor blocks to generate *X(i + 1)* and *Z(i + 1)*. At the end of the architecture, the desired output (*Z(i + 1)*) is produced in two different states. For this reason, the value of *X(i)* and the zero number are compared, and then one of the states will be selected. It is noticeable that the *Z* variable is equal to multiplying the *X* by the *Y* variable. Additionally, the CORDIC structure needs *16* iterations to perform completely and approximates the multiplication. Furthermore, the total iteration is *32* to produce $V^{3}$ in the CORDIC unit of Fig 12.

## Hardware results and discuss

**Fig 14:**
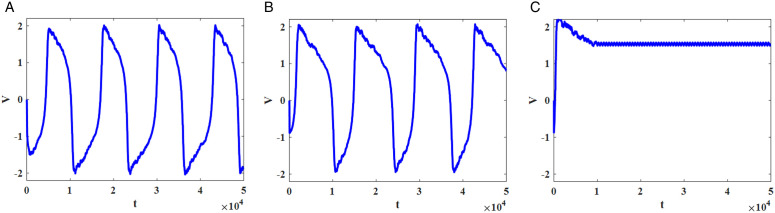
Output waveforms of the proposed FHN hardware implementation on the FPGA platform based on different input currents. (a) I_in_ = 0.5 mA. (b) I_in_ = 1 mA. (c) I_in_ = 2 mA.

This section consists of results of the hardware implementation using the CORDIC-based FHN model, which was described in the previous sections. Hardware Description Language (VHDL) is applied to implement the scheduling diagrams of the CORDIC and original FHN models in order to validate the modified model. After that, the VHDL code is synthesized on a Virtex-4 FPGA platform in Xilinx ISE. Therefore, the regular tonic pattern of the proposed model in three cases are illustrated in Fig 14. The figure shows that the CORDIC model correctly reproduces the similar behavior of the original model. According to the equations of the original model, it utilizes some multiplications, divisions, and a nonlinear term to realize the FHN model.

The main challenge of hardware implementation on FPGA is developing models without employing any multipliers because the device has a limited number of multipliers. If the original model is implemented on the FPGA platform, the results should be included in ineffective hardware implementation with low speed, high hardware resources, and raised power consumption. Hence, the suggested model is just employing ADD and Shift operations instead of the aforementioned factors in the original model. It should be noted that there is a trade-off between hardware cost and accuracy when the CORDIC algorithm is realized on digital hardware because the CORDIC algorithm is an iterative process. Table 6 shows the hardware implementation results of the suggested method in ISE software. From the table, the CORDIC-based FHN model is developed without utilizing any multipliers, and also the model’s maximum frequency is acceptable in comparison to some previous research. Therefore, by applying the CORDIC-based FHN model, it is possible to develop multiplierless hardware with high accuracy and acceptable speed. On the other hand, the power consumption, which is a significant concern for hardware design, is measured on FPGA devices by using the Xilinx XPower analysis. Table 7 displays the results of the hardware implementation of the CORDIC-based FHN model on ISE software by employing the XPower analysis. Dynamic, static, and leakage component values are calculated by the tool and reported in the table. The result depicts the power consumption for the suggested model in [20] as *295.49 mW*, while for our work, that is *182.41 mW*. It is noticeable that some factors haven’t been measured or reported by the aforementioned literature. As a consequence, the low-cost hardware implementation is available by applying the CORDIC-based FHN model in comparison with the original. Furthermore, the power consumption of our work is less than all proposed methods, so the suggested model is appropriate to design low-power hardware on FPGA. Based on the simulation errors, which were indicated in Table 2, and the results of hardware implementation, we introduce new criteria to completely compare the presented FHN models in two aspects of

**Table 6:** The device utilization of Xilinx Virtex-4 and the used percentage of basic elements for implementation of the proposed FHN model.

Model	DSPs	Slices	Slice Flip Flops	4-In-LUTs	Frequency (MHZ)
Proposed FHN	0 (0%)	478 (8%)	360 (3%)	849 (7%)	232.156

**Table 7:** Power supply summary of the FHN model implementation on the Virtex-4 FPGA platform.

	Total	Dynamic	Static	Leakage
Supply Power (mW)	182.41	15.72	166.69	167

Aspects of digital implementation, including maximum frequency and power consumption. For this reason, two cost functions (CF) are presented, which are given as:


$$CF_{1}=\frac{MAE}{\begin{array}{ccc} {}*20c & Freq & \end{array}}$$ (25)

$$CF_{2}=\frac{MAE}{\begin{array}{ccc} {}*20c & Freq & \end{array}}\times Power$$ (26)

Where *MAE* is a simulation error that is calculated in Table 2, and also the Freq and Power denote the maximum frequency and power consumption, which are obtained in hardware implementation. Considering both cost functions, the minimum values of them are our desired states. In other words, a rise in Freq and a decrease in *MAE* or power can reduce the cost functions. Table 8 illustrates the results of employing the cost functions for all FHN works. From this table, it is clear that both cost functions for our work are less than other proposed methods. Therefore, the results of Table 8 indicate that the high-accuracy and low-power digital implementation is achieved when the CORDIC-based FHN model is applied. The main reason to propose our model is that the CORDIC algorithm hasn’t been implemented for the FHN model. Although the work in [20] presented a CORDIC approach, which used the CORDIC IP-Core of FPGA, to approximate the nonlinear term of the FHN model, we implement the CORDIC-based FHN model by only using Add and Shift operations. Due to the iterative process of the CORDIC algorithm, it was anticipated that the hardware resource of developing the digital system on FPGA wasn’t less than some methods. Nevertheless, the accuracy of the suggested model is more acceptable than other FHN models because the CORDIC function approximates the results by iterating the algorithm. Moreover, the algorithm is suitable for designing low-power digital hardware as it just utilizes ADD and Shift operations instead of nonlinear terms. Thus, the hardware implementation of the CORDIC-based FHN model is an appropriate option for digital design since it is low-power and highly precise.

**Table 8:** Cost functions calculations for proposed FHN model and other presented models.

References	MAE	Frequency (MHZ)	Power (mW)	CF_1_	CF_2_
[42]	0.03	175	NA	$171\cdot 43\times 10^{-12}$	--
[20]	0.015	320	295.49	$46\cdot 88\times 10^{-12}$	$13\cdot 85\times 10^{-12}$
[45]	0.0034	377	NA	$9\cdot 02\times 10^{-12}$	--
**Our work**	0.00083	232.156	182.41	$3\cdot 57\times 10^{-12}$	$0\cdot 652\times 10^{-12}$

## Conclusion

A multiplierless FHN model has been presented and implemented on an FPGA by employing the CORDIC algorithm in this research. The applied CORDIC method benefits from adders and shifters to remove multiplications. The proposed CORDIC-based FHN neuron, by selecting the suitable number of iterations, accurately mimics the dynamical behavior of the original neural model, providing cost-efficient digital hardware. Additionally, we employed a sinusoidal input current to stimulate the FHN model, so chaotic behaviors were seen in certain values of the input parameters for both the original and proposed digital FHN models. According to the results of the hardware implementation on the FPGA platform, the low-power realization of the proposed model with high accuracy was achievable. It should be mentioned that we have introduced two cost functions for more assessment between the works in the area. The cost functions consist of maximum frequency, power consumption, and error. The results of cost functions proved that the FPGA-based digital implementation of the suggested FHN model is low-power and high-accuracy with acceptable speed. Therefore, due to the low power consumption, low error, and high frequency, the presented hardware is able to be effective and useful in various applications, including the modeling of learning of the nervous system based on nonlinear and chaotic behaviors.

## Supporting information

S1 File Software_code. (ZIP)

S2 File Hardware_code. (ZIP)

## References

[1] Schuman CD, Potok TE, Patton RM, Birdwell JD, Dean ME, Rose GS. A survey of neuromorphic computing and neural networks in hardware. arXiv preprint. 2017. doi: 10.48550/arXiv.1705.06963

[2] Ahmed K. Brain-inspired spiking neural networks. Biomimetics. IntechOpen; 2020.

[3] Yamazaki K, Vo-Ho V-K, Bulsara D, Le N. Spiking neural networks and their applications: a review. Brain Sci. 2022;12(7):863. doi: 10.3390/brainsci12070863 35884670 PMC9313413

[4] Ghosh-Dastidar S, Adeli H. Third generation neural networks: Spiking neural networks. Advances in computational intelligence. Berlin, Heidelberg: Springer Berlin Heidelberg; 2009. pp. 167–78.

[5] Diehl PU, Cook M. Unsupervised learning of digit recognition using spike-timing-dependent plasticity. Front Comput Neurosci. 2015;9:99. doi: 10.3389/fncom.2015.00099 26941637 PMC4522567

[6] Nazari S, faez K. Spiking pattern recognition using informative signal of image and unsupervised biologically plausible learning. Neurocomputing. 2019;330:196–211. doi: 10.1016/j.neucom.2018.10.066

[7] Gutierrez-Galan D, Dominguez-Morales JP, Perez-Peña F, Jimenez-Fernandez A, Linares-Barranco A. NeuroPod: a real-time neuromorphic spiking CPG applied to robotics. Neurocomputing. 2020;381:10–9.

[8] Aboumerhi K, Güemes A, Liu H, Tenore F, Etienne-Cummings R. Neuromorphic applications in medicine. J Neural Eng. 2023;20(4):041004. doi: 10.1088/1741-2552/aceca3 37531951

[9] Hodgkin AL, Huxley AF. A quantitative description of membrane current and its application to conduction and excitation in nerve. J Physiol. 1952;117(4):500. 12991237 10.1113/jphysiol.1952.sp004764 PMC1392413

[10] Morris C, Lecar H. Voltage oscillations in the barnacle giant muscle fiber. Biophys J. 1981;35(1):193–213. 7260316 10.1016/S0006-3495(81)84782-0 PMC1327511

[11] Wilson HR. Simplified dynamics of human and mammalian neocortical neurons. J Theor Biol. 1999;200(4):375–88. doi: 10.1006/jtbi.1999.1002 10525397

[12] Rose RM, Hindmarsh JL. The assembly of ionic currents in a thalamic neuron. I. The three-dimensional model. Proc R Soc Lond B Biol Sci. 1989;237(1288):267–88. doi: 10.1098/rspb.1989.0049 2571154

[13] Fitzhugh R. Impulses and physiological states in theoretical models of nerve membrane. Biophys J. 1961;1(6):445–66. doi: 10.1016/s0006-3495(61)86902-6 19431309 PMC1366333

[14] Izhikevich EM. Simple model of spiking neurons. IEEE Trans Neural Netw. 2003;14(6):1569–72. doi: 10.1109/tnn.2003.820440 18244602

[15] Gerstner W, Kistler WM. Spiking neuron models: Single neurons, populations, plasticity. Cambridge University Press; 2002.

[16] Brette R, Gerstner W. Adaptive exponential integrate-and-fire model as an effective description of neuronal activity. J Neurophysiol. 2005;94(5):3637–42. doi: 10.1152/jn.00686.2005 16014787

[17] Hedayatpour MA, Karami MA, Shamsi J. Implementation of Izhikevich neuron based on stochastic computing using a novel inspired Omega‐Flip stochastic number generator. Int J Circuit Theory Apps. 2022;50(9):3104–18. doi: 10.1002/cta.3322

[18] Soleimani H, Ahmadi A, Bavandpour M. Biologically inspired spiking neurons: piecewise linear models and digital implementation. IEEE Trans Circuits Syst I. 2012;59(12):2991–3004. doi: 10.1109/tcsi.2012.2206463

[19] Rajasekar S, Lakshmanan M. Bifurcation, chaos and suppression of chaos in FitzHugh-Nagumo nerve conduction model equation. J Theor Biol. 1994;166(3):275–88. doi: 10.1006/jtbi.1994.1025 8159015

[20] Haghiri S, Yahya SI, Rezaei A, Ahmadi A. Multiplierless implementation of Fitz-Hugh Nagumo (FHN) modeling using CORDIC approach. IEEE Trans Emerg Top Comput Intell. 2024;8(1):279–87. doi: 10.1109/tetci.2023.3300176

[21] Xu Q, Chen X, Chen B, Wu H, Li Z, Bao H. Dynamical analysis of an improved FitzHugh-Nagumo neuron model with multiplier-free implementation. Nonlin Dyn. 2023;111(9):8737–49.

[22] Hazan A, Ezra Tsur E. Neuromorphic analog implementation of neural engineering framework-inspired spiking neuron for high-dimensional representation. Front Neurosci. 2021;15:627221. 33692670 10.3389/fnins.2021.627221 PMC7937893

[23] Rathi N, Chakraborty I, Kosta A, Sengupta A, Ankit A, Panda P, et al. Exploring neuromorphic computing based on spiking neural networks: algorithms to hardware. ACM Computing Surveys. 2023;55(12):1–49.

[24] Wahid SA, Asad A, Mohammadi F. A survey on neuromorphic architectures for running artificial intelligence algorithms. Electronics. 2024;13(15):2963.

[25] Furber S. Digital neuromorphic technology: current and future prospects. Natl Sci Rev. 2023;11(5). doi: 10.1093/nsr/nwad283 PMC10989295 38577676

[26] Kuon I, Tessier R, Rose J. FPGA architecture: survey and challenges. FNT Electron Design Autom. 2008;2(2):135–253. doi: 10.1561/1000000005

[27] Alkhafaji FSM, Hasan WZW, Isa MM, Sulaiman N. Robotic controller: ASIC versus FPGA–a review. J Comput Theor Nanosci. 2018;15(1):1–25. doi: 10.1166/jctn.2018.7119

[28] Han J, Li Z, Zheng W, Zhang Y. Hardware implementation of spiking neural networks on FPGA. Tinshhua Sci Technol. 2020;25(4):479–86. doi: 10.26599/tst.2019.9010019

[29] Pham QT, Nguyen TQ, Hoang PC, Dang QH, Nguyen DM, Nguyen HH. A review of SNN implementation on FPGA. In: 2021 International Conference on Multimedia Analysis and Pattern Recognition (MAPR). 2021. pp. 1–6.

[30] Amiri M, Nazari S, Faez K. Digital realization of the proposed linear model of the Hodgkin‐Huxley neuron. Int J Circ Theory Apps. 2019;47(3):483–97. doi: 10.1002/cta.2596

[31] Das MK, Saha LM. Chaotic dynamics and complexity in real and physical systems. Advances in Dynamical Systems Theory, Models, Algorithms and Applications. 2021. pp. 1–27.

[32] Rabinovich MI, Abarbanel HD. The role of chaos in neural systems. Neuroscience. 1998;87(1):5–14. 9722138 10.1016/s0306-4522(98)00091-8

[33] Wang Q, Lu Q, Chen G, Duan L. Bifurcation and synchronization of synaptically coupled FHN models with time delay. Chaos Solitons Fractals. 2009;39(2):918–25.

[34] Manchein C, Santana L, da Silva RM, Beims MW. Noise-induced stabilization of the FitzHugh–Nagumo neuron dynamics: multistability and transient chaos. Chaos: An Interdiscip J Nonlin Sci. 2022;32(8). doi: 10.1063/5.0086994 36049914

[35] Cebrián-Lacasa D, Parra-Rivas P, Ruiz-Reynés D, Gelens L. Six decades of the FitzHugh–Nagumo model: a guide through its spatio-temporal dynamics and influence across disciplines. Phys Rep. 2024;1096:1–39. doi: 10.1016/j.physrep.2024.09.014

[36] Huang G, Zhou S, Zhu R, Wang Y, Chai Y. Stability and complexity evaluation of attractors in a controllable piezoelectric Fitzhugh-Nagumo circuit. Chaos Solitons Fractals. 2024;182:114802.

[37] Strogatz SH. Nonlinear dynamics and chaos: with applications to physics, biology, chemistry, and engineering. Boca Raton: CRC Press; 1994.

[38] Salas-Paracuellos L, Alba L, Villacorta-Atienza JA, Makarov VA. FPGA implementation of a modified FitzHugh-Nagumo neuron based causal neural network for compact internal representation of dynamic environments. In: Bioelectronics, Biomedical, and Bioinspired Systems V; and Nanotechnology V. Vol. 8068. SPIE; 2011. pp.170–8.

[39] Adon NA, Mahmud F, Jabbar MH, Othman N. FPGA-in-the-loop co-simulation of reentrant arrhythmia mechanism in one dimensional (1D) ring-shaped based on FitzHugh-Nagumo model. In: 2014 IEEE International Conference on Control System, Computing and Engineering (ICCSCE 2014). IEEE; 2014. pp. 288–93.

[40] Nouri M, Karimi GhR, Ahmadi A, Abbott D. Digital multiplierless implementation of the biological FitzHugh–Nagumo model. Neurocomputing. 2015;165:468–76. doi: 10.1016/j.neucom.2015.03.084

[41] Hayati M, Nouri M, Haghiri S, Abbott D. A digital realization of astrocyte and neural glial interactions. IEEE Trans Biomed Circ Syst. 2015;10(2):518–29. 10.1109/TBCAS.2015.2450837 26390499

[42] Zahedi A, Haghiri S, Hayati M. Multiplierless digital implementation of time-varying FitzHugh–Nagumo model. IEEE Trans Circ Syst I. 2019;66(7):2662–70. doi: 10.1109/tcsi.2019.2899361

[43] Xu Q, Zhu D. FPGA-based experimental validations of electrical activities in two adjacent FitzHugh–Nagumo neurons coupled by memristive electromagnetic induction. IETE Technical Review. 2021;38(6):563–77.

[44] Korkmaz N, Şivga B. The FPGA-based realization of the electromagnetic effect defined FitzHugh-Nagumo neuron model. Chaos Theory Appl. 2022;4(2):88–93. doi: 10.51537/chaos.1101581

[45] Ge Y, Liu R, Zhang G, Daoud MSH, Zhang Q, Huang X, et al. High-matching and low-cost realization of the FHN neuron model on reconfigurable FPGA Board. IEEE Trans Biomed Circuits Syst. 2024;18(2):451–9. doi: 10.1109/tbcas.2023.3337335 38019637

[46] Nazari S, Faez K, Amiri M, Karami E. A digital implementation of neuron–astrocyte interaction for neuromorphic applications. Neural Netw. 2015;66:79–90. 25814323 10.1016/j.neunet.2015.01.005

[47] Andraka R. A survey of CORDIC algorithms for FPGA based computers. In: Proceedings of the 1998 ACM/SIGDA sixth international symposium on Field programmable gate arrays. 1998. pp. 191–200.

[48] Meher PK, Valls J, Tso-Bing J, Sridharan K, Maharatna K. 50 years of CORDIC: algorithms, architectures, and applications. IEEE Trans Circuits Syst I. 2009;56(9):1893–907. doi: 10.1109/tcsi.2009.2025803

[49] Lakshmi B, Dhar AS. CORDIC architectures: a survey. VLSI Design. 2010;2010:1–19. doi: 10.1155/2010/794891

